# Effects of Varying Acupuncture Manipulations at ST36 (Zusanli) on Gastric Electrical Frequency and Amplitude in Bradygastria Rabbits

**DOI:** 10.1155/2020/9357120

**Published:** 2020-03-09

**Authors:** Kang Wang, Yuan Xu, Yan Niu, Yangyang Liu, Hezheng Lai, Zhifang Xu, Kuo Zhang, Yongming Guo, Yi Guo

**Affiliations:** ^1^Dongfang Hospital, Beijing University of Traditional Chinese Medicine, Beijing 100078, China; ^2^Experimental Acupuncture-Moxibustion Research Center, Tianjin University of Traditional Chinese Medicine, Tianjin 301617, China; ^3^Chinese Medicine Centre, Collaboration Between Beijing University of Chinese Medicine and Western Sydney University, Campbelltown 2560, NSW, Australia; ^4^School of Acupuncture and Massage, Tianjin University of Traditional Chinese Medicine, Tianjin 301617, China; ^5^The 3rd Affiliated Hospital of Beijing University of Traditional Chinese Medicine, Beijing 100029, China; ^6^NICM Health Research Institute, Western Sydney University, Westmead 2145, NSW, Australia; ^7^Academy of Medical Engineering and Translational Medicine, Tianjin University, Tianjin 300072, China; ^8^School of Traditional Chinese Medicine, Tianjin University of Traditional Chinese Medicine, Tianjin 301617, China

## Abstract

**Objective:**

To observe the effects of different manual acupuncture (MA) manipulation on gastric electrical amplitude and frequency for noradrenaline-induced bradygastria in rabbits.

**Methods:**

A total of 60 rabbits were randomly allocated into six groups: four MA manipulation groups; reinforcing by twisting the manipulation group (FTG), reducing by twisting the manipulation group (RTG), reinforcing by lifting and thrusting the manipulation group (FLG), and reducing by lifting and thrusting the manipulation group (RLG), a control group (CG), and a model group (MG). The total treatment time length was 45 minutes. The bradygastria was induced via administration of noradrenaline via the marginal ear vein of the rabbits at 5 minutes from baseline, and the bradygastria model was established at 12 minutes from baseline. The rabbits in the four MA manipulation groups received different stimulation parameters at ST36 (Zusanli) for a duration of 3 minutes in accordance with their respective group allocation. The needles were then retained without further manipulation for a further 25 minutes. Gastric electrical amplitude and frequency were recorded using a data acquisition system (Biopac System MP150) at five different time points: baseline (for a duration of 5 minutes), after the bradygastria model was established at 12 minutes from baseline (for a duration of 5 minutes), during MA manipulation commencing at 17 minutes from baseline (for a duration of 3 minutes), 5 minutes after MA manipulation at 25 minutes from baseline (for a duration of 5 minutes), and at 20 minutes following MA manipulation at 40 minutes from baseline (for a duration of 5 minutes).

**Results:**

After noradrenaline induction, gastric electrical frequency levels in MA and MG groups were significantly decreased compared to the CG group (*P* < 0.05). However, there were no significant changes to gastric electrical amplitude (*P* < 0.05). However, there were no significant changes to gastric electrical amplitude (*P* < 0.05). However, there were no significant changes to gastric electrical amplitude (*P* < 0.05). However, there were no significant changes to gastric electrical amplitude (*P* < 0.05). However, there were no significant changes to gastric electrical amplitude (*P* < 0.05). However, there were no significant changes to gastric electrical amplitude (

**Conclusion:**

All four variations of MA manipulations have a recovery effect on the gastric electrical frequency of rabbits with bradygastria. In particular, results indicated that FTG, RTG, and FLG at ST36 may have a regular and significant recovery trend through the whole process of the acupuncture intervention.

## 1. Introduction

With a history of more than 2000 years, acupuncture in traditional Chinese medicine (TCM) is a practice that has accumulated rich theoretical and clinical experiences [1] and has been widely used around the world. It has been proven to treat a wide range of diseases and conditions [2]. However, acupuncture has yet to be accepted as a treatment in conventional medicine, owing to the present lack of understanding of the scientific mechanisms that explain its efficacy [3]. Acupuncture therapy is not simply the insertion of needles into points along the body's meridian pathways. There are many MA manipulation techniques, such as lifting, thrusting, twisting, etc, which may be used in a variety of combinations to achieve different therapeutic effects. Acupuncture manipulation and stimulation frequency play a major role in the delivery of specific therapeutic effects [4, 5]. Most research has focused on acupoint specificity, less on exploring the effect of acupuncture manipulation [6]. Several studies have confirmed that different acupuncture manipulation techniques produce different bioelectric effects: acupuncture manipulation can cause the excitement of receptors in afferent nerves affecting the nervous system, stimulate signals affecting the central nervous system's integration and alternation of electrical signals, and affect microcirculation blood perfusion to the local skin and temperature on the acupoint's area [7–9]. However, further research is necessary to establish the mechanisms behind the treatment effect of acupuncture.

With improvements to people's standard of living, changes in eating habits and diversification of nutrition, the prevalence of gastrointestinal (GI) diseases has risen substantially in recent years. In 2004, GI diseases were reported to affect an estimated 60 to 70 million United States citizens, contributing to approximately $142 billion in direct and indirect costs [10]. Gastric motility disorders make up 30% to 40% of GI diseases. Bradygastria is a common gastric motility disorder, with clinical presentations of upper abdominal distension, belching, nausea, vomiting, heartburn, and insomnia [11]. It can occur as a primary condition or a secondary condition related to other diseases, such as systemic sclerosis [12], and nausea and vomiting after intensive chemotherapy [13]. Acupuncture is an alternative therapy beneficial for the management of chemotherapy-induced nausea, peptic ulcer disease, and postoperative ileus, as well as other gastrointestinal functional disorders such as irritable bowel syndrome [14], which may be induced by bradygastria.

Similar to the heart, the stomach has an intrinsic pacemaker that generates gastric myoelectrical activity comprising slow waves and spikes. It determines the maximum frequency and propagation of gastric contractions [15]. Therefore, observing changes in gastric electric frequency and amplitude can be an effective method to diagnose abnormal activity of smooth muscle. This study aimed to investigate four variations of MA manipulations at ST36 (Zusanli) and the respective effects on gastric electrical amplitude and frequency in noradrenaline-induced bradygastria in rabbits.

## 2. Methods

### 2.1. Animals

Experiments were performed on 60 healthy adult rabbits; 30 males and 30 females (2 ± 0.2 kg) (Animal license number SCXK Jin 2009-0001). Before the experiment, all rabbits were adaptively fed for 7 days. All animals had access to food and water ad libitum in temperature (23 ± 1°C)- and humidity (50 ± 5%)-controlled rooms with 12-hour light-dark cycles. The rabbits fasted 14–18 hours before the experiment. All MA manipulations and procedures were carried out in accordance with the Guide for Care and Use of Laboratory Animals issued by USA National Institutes of Health and were approved by the Animal Ethics Committee in Tianjin University of Traditional Chinese Medicine in China.

### 2.2. Bradygastria Models

Bradygastria was induced in the rabbits by injection of 30 ml noradrenaline concentrate solution (containing 0.6 mg/kg of noradrenaline bitartrate injection (Tianjin Golden Crown Amino Acid Co., Ltd., Tianjin, China) and 0.9% normal saline) at constant speed (0.4 mg/kg·h) into the marginal ear vein [16].

### 2.3. Gastric Electrical Measurement

**Figure 1:**
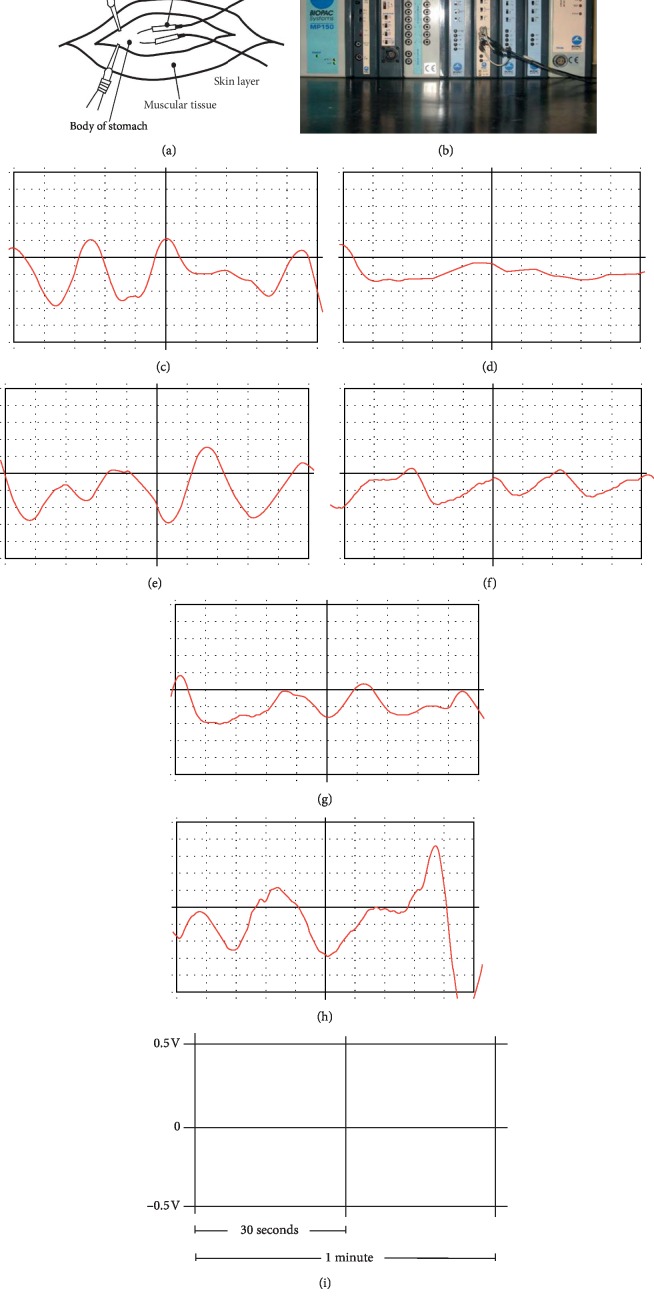
The detail of gastric electrical signal measurement. (a) The location of two EL450 stainless steel needle electrodes (0.5 cm gap). (b) The data acquisition system MP150, BIOPAC Systems. (c) The electrogastrography (EGG) of the control group. (d) The EGG of the model group. (e) The EGG of reinforcing by twisting manipulation. (f) The EGG in reducing by twisting manipulation. (g) The EGG of reinforcing by lifting and thrusting manipulation. (h) The EGG of reducing by lifting and thrusting manipulation. (i) All EGG graphs above were shown in the length of 1 minute.

**Figure 2:**
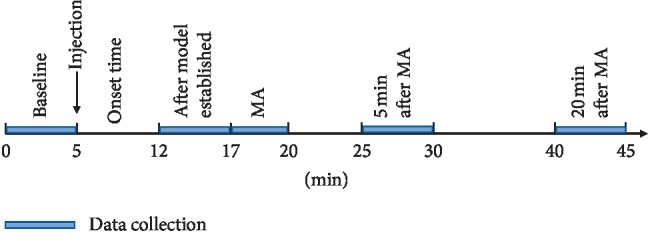
Timeline of gastric electrical recording and manual acupuncture treatment at ST36.

Animals were anaesthetized by marginal ear-vein injection of 20% urethane (5 ml/kg), and the anesthetic effect can normally last for one hour. 4% heparin sodium in 0.9% saline amounting to a total 0.6 ml was used to seal the tube in order to prevent the internal coagulation of the indwelling needle. A 4 cm vertical incision was made at 1 cm along the subxiphoid and at 2 cm on the right side of the median line of the abdomen. The skin, subcutaneous tissue, muscle, and peritoneum were cut by each respective layer to expose the gastric body of the rabbit. In order to prevent that the exposed stomach becoming too cold, we use an electric heater to maintain the room temperature at 25 degrees and covered wound by medical gauze. Gastric electrical signals were recorded by data acquisition system (MP150, BIOPAC Systems, Inc., Goleta, CA, USA). Two EL450 stainless steel needle electrodes (0.5 cm gap) were inserted into the serosal layer of the rabbit's gastric body in the direction consistent with the long axis of the stomach (Figure 1(a)). The needle electrodes were connected with an electrogastrogram (EGG) 100C module (Figure 1(b)). All data were collected at acquisition parameters: GAIN: 1000, high pass: 1 Hz, and low pass: 0.005 Hz. The amplitudes and frequency of the gastric electrical signals were collected at baseline, after modelling, MA, 5 mins after MA, and 20 mins after MA (Figure 2). 5 mins after the MA period was aimed to observe the instant acupuncture effect, and 20 mins after the MA period was aimed to observe the normal acupuncture effect.

### 2.4. Acupuncture Treatments

**Figure 3:**
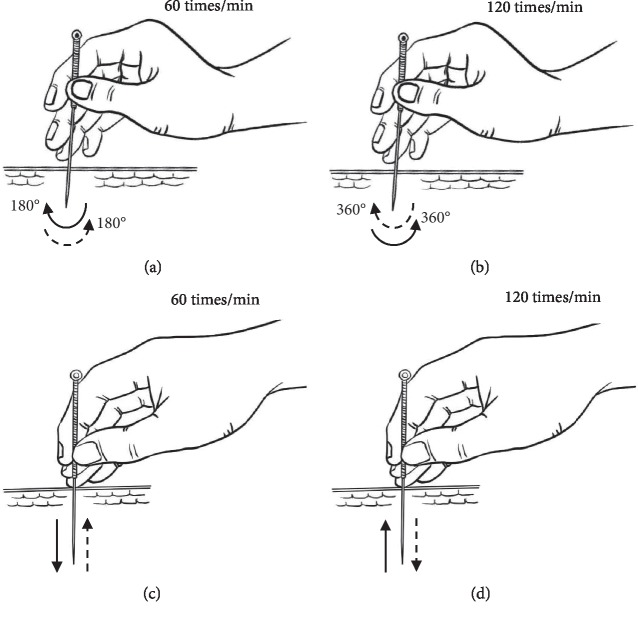
Parameters of the four different MA manipulations. (a) Reinforcing by twisting the needle. (b) Reducing by twisting the needle. (c) Reinforcing by lifting and thrusting the needle. (d) Reducing by lifting and thrusting manipulation.

Acupuncture was performed on the left side ST36 (Zusanli) of the rabbits using disposable acupuncture needles (0.20 mm × 25 mm, Zhongyan Taihe Medical Instruments Co. Ltd., Beijing, China). ST36 is located between the tibia and fibula, at approximately 12 mm beneath the fibulae capitulum and 1 cm behind the tibial crest [17]. The needles were inserted 5–7 mm deep into the skin, while taking care to avoid nerves and blood vessels. In accordance with traditional acupuncture theory, in particular the use of “light manipulations to reinforce and heavy manipulations to reduce,” after deqi was achieved, ST36 was stimulated with four kinds of manipulations in accordance to the four respective MA groups (Figure 3): (a) Reinforcing by twisting the needle: rotating the needle with the thumb forward forcefully with large amplitude (frequency: 60 times/min, rotation angle: 180°). (b) Reducing by twisting the needle: rotating the needle with the thumb backward forcefully with large amplitude (frequency: 120 times/min, rotation angle: 360°). (c) Reinforcing by lifting and thrusting the needle: lifting the needle gently and slowly, while thrusting the needle heavily and rapidly (frequency: 60 times/min, direction: perpendicular to the skin). (d) Reducing by lifting and thrusting manipulation: lifting the needle forcefully and rapidly while thrusting the needle gently and slowly (frequency: 120 times/min, direction: perpendicular to the skin). All of the manipulations were performed by the chief researcher (KW), who was a qualified master student and obtained the qualification of national medical doctor.

### 2.5. Experimental Procedure

Sixty rabbits were randomly divided into six groups: reinforcing by twisting the needle group (FTG), reducing by twisting the needle group (RTG), reinforcing by lifting and thrusting the needle group (FLG), reducing by lifting and thrusting the manipulation group (RLG), the control group (CG), and the model group (MG). A baseline reading for gastric electrical frequency and amplitude were recorded using the electrogastrogram over a five-minute time period. In the MA and MG groups, 30 ml noradrenaline concentrate solution was injected continuously into the marginal ear vein of rabbits at a constant rate. In the CG, 0.9% saline was injected at the same rate. Bradygastria occurred within a timeframe of 180 to 420 seconds following noradrenaline injection and the bradygastria model determined at twelve minutes from baseline. The gastric electricity frequency and amplitude of the bradygastria model were recorded during the subsequent five-minute time period. Thereafter, acupuncture needles were inserted, and in the MA groups, manipulation was performed for a duration of three minutes. Gastric electrical frequency and amplitude were recorded during the three-minute time period of continuous MA manipulation. After five minutes had elapsed following the cessation of MA manipulation, gastric electrical frequency and amplitude were again recorded over a five-minute time period, and a final record was made at twenty minutes following MA manipulation, over a five-minute time period (Figure 2).

### 2.6. Data Preprocessing and Analysis

Biopac System MP150 was used to capture real-time dynamic monitoring of the gastric electric frequency and amplitude and analysis data. Finite impulse response (FIR) was used to filter the gastric electrical data. The parameters were high frequency: 0.167 Hz, low frequency: 0.005 Hz, and number of coefficients: 2400. Results for the gastric electric frequency and amplitude for the respective groups were calculated by determining the average value recorded at each of the five time points. All data were presented as mean ± SD. Multiple measurements at different time points were analyzed by repeated-measures analysis of variance (ANOVA). LSD method was used if data were determined as consistent with the homogeneity test of variance, while Dunnett's T3 method was used if data were determined as inconsistent with the homogeneity test of variance, as established using SPSS 19.0. *P* < 0.05 or *P* < 0.01 was regarded as statistically significant.

## 3. Results

### 3.1. Gastric Electrical Amplitude

**Figure 4:**
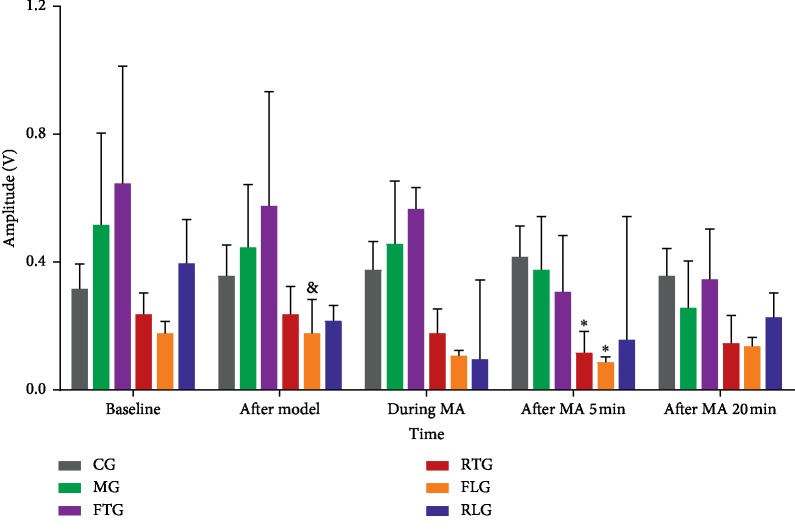
Effect of different MA manipulation on gastric electrical amplitude for bradygastria rabbits. Control group (CG), model group (MG), reinforcing by twisting the needle group (FTG), reducing by twisting the needle group (RTG), reinforcing by lifting and thrusting the needle group (FLG), and reducing by lifting and thrusting the manipulation group (RLG). The results are expressed as the mean ± SD. *N* = 10 per group. ^*∗*^*P* < 0.05 vs. CG, ^&^*P* < 0.05 vs. FTG.

There was no statistically significant difference between the comparison of gastric electrical amplitude in the CG and MG at all five time points.The results indicated that noradrenaline had little effect on the changes of gastric electrical amplitude (*P* > 0.05). There was no statistically significant difference between the four manipulation groups and MG at all five time (*P* > 0.05). Compared with 0.65 ± 0.91 V in the FTG group, gastric electrical amplitude in the FLG was decreased to 0.18 ± 0.11 V at baseline (*P* < 0.05). Compared with 0.42 ± 0.24 V in the CG group, gastric electrical amplitude was decreased to 0.12 ± 0.16 V in RTG and 0.09 ± 0.04 V in FLG (*P* < 0.05), at the time point of 5 minutes after MA manipulation (Figure 4).

### 3.2. Gastric Electrical Frequency

Compared with CG, gastric electrical frequency in other groups were no statistical differences on baseline. The gastric electrical frequency in the CG did not significantly change over time.

After bradygastria model was established, the gastric electrical frequency in MG was decreased to 3.11 ± 0.60 times/min (*P* < 0.01), 2.82 ± 0.33 times/min in FTG (*P* < 0.01), 3.74 ± 1.11 times/min in RTG (*P* < 0.05), 3.30 ± 0.87 times/min in FLG (*P* < 0.01), and 3.23 ± 0.69 times/min in RLG (*P* < 0.01), when compared with 4.43 ± 0.62 times/min in CG.

During the manipulation acupuncture (MA), the gastric electrical frequencies in MG were decreased to 3.00 ± 0.66 times/min (*P* < 0.01), when compared with 4.32 ± 0.43 times/min in CG. The gastric electrical frequencies were increased to 3.56 ± 1.00 times/min in FTG (*P* > 0.05), 3.89 ± 0.87 times/min in RTG (*P* < 0.05), 4.15 ± 1.36 times/min in FLG (*P* < 0.01), and 4.15 ± 0.93 times/min in RLG (*P* < 0.01), when compared with 3.00 ± 0.66 times/min in MG.

During after MA 5 min, the gastric electrical frequencies in MG were decreased to 3.65 ± 0.92 times/min (*P* > 0.05), when compared with 4.30 ± 0.42 times/min in CG. The gastric electrical frequencies were increased to 4.55 ± 1.38 times/min in FTG (*P* > 0.05), 5.33 ± 1.00 times/min in RTG (*P* < 0.01), and 5.20 ± 1.06 times/min in FLG (*P* < 0.01), but decreased to 3.64 ± 1.11 times/min in RLG (*P* > 0.05), when compared with 3.65 ± 0.92 times/min in MG.

During after MA 20 min, the gastric electrical frequencies in MG were decreased to 3.49 ± 0.70 times/min (*P* < 0.05), when compared with 4.53 ± 0.75 times/min in CG. The gastric electrical frequencies were increased to 4.38 ± 1.27 times/min in FTG (*P* < 0.05), 5.22 ± 0.84 times/min in RTG (*P* < 0.01), 4.40 ± 1.08 times/min in FLG (*P* < 0.05), and 4.29 ± 0.86 times/min in RLG (*P* > 0.05), when compared with 3.49 ± 0.70 times/min in MG.

**Figure 5:**
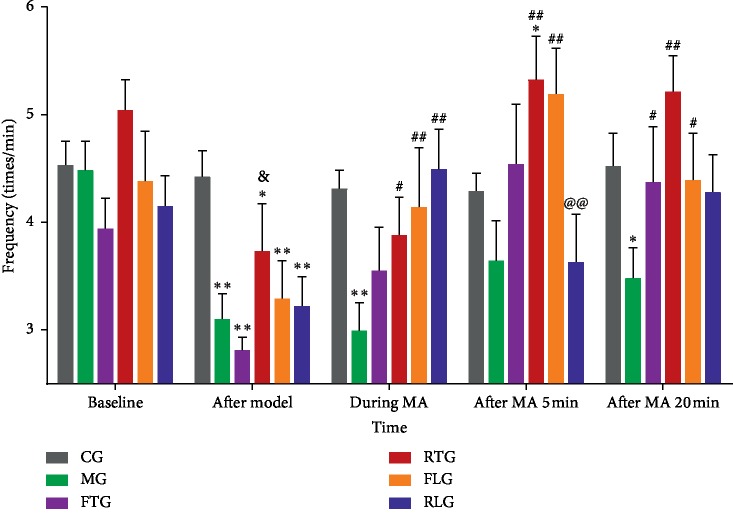
Effect of different MA manipulation on gastric electrical frequency for bradygastria rabbits. Control group (CG), model group (MG), reinforcing by twisting the needle group (FTG), reducing by twisting the needle group (RTG), reinforcing by lifting and thrusting the needle group (FLG), and reducing by lifting and thrusting the manipulation group (RLG). The results are expressed as the mean ± SD. *N* = 10 per group. ^*∗∗*^*P* < 0.01, ^*∗*^*P* < 0.05 vs. CG, ^##^*P* < 0.01, ^#^*P* < 0.05 vs. MG, ^&^*P* < 0.05 vs. FTG, ^@@^*P* < 0.01 vs. FLG.

In addition, after bradygastria model was established, the gastric electrical frequencies in RTG were increased to 3.74 ± 1.11 times/min compared with 2.82 ± 0.33 times/min in FTG (*P* < 0.05). During after MA 5 min, the gastric electrical frequencies in RTG were decreased to 3.64 ± 1.11 times/min compared with 5.20 ± 1.06 times/min in FLG (*P* < 0.01) and increased 5.33 ± 1.00 times/min in RTG compared with 4.30 ± 0.42 times/min in CG (*P* < 0.05) (Figure 5).

## 4. Discussion

Gastric electrical stimulation can accurately reflect gastric function in real time, thereby providing an objective bioelectrical index for the physiological and pathological changes of the stomach. Gastric electrical activity data have proven valuable in the study of gastric diseases, as well as in the diagnosis, treatment, and curative evaluation of epigastric pain [18, 19]. Acupuncture has been shown to have a regulatory effect on gastric electrical stimulation [20, 21]. These acupunctural effects suggest a good connection with the theory of acupoints-viscera correlation and relative specificity of acupoints [22, 23]. However, a lack of research in the effects of the different manipulations themselves, in acupuncture, remains. The manipulation of acupuncture is a key component in the treatment process, and different manipulations may result in significantly different outcomes [24]. Acupoint ST36 of the stomach meridian of Foot-Yangming is an important acupoint for the regulation of gastric motility, as shown in several studies [25, 26].

The present study confirmed that different manipulations on ST36 had varying effects on gastric electrical amplitude and frequency. Noradrenaline had little effect on the changes of gastric electrical amplitude (*P* > 0.05). However, at five minutes after MA manipulation, the amplitude of gastric electrical stimulation in RTG and FLG was decreased, when compared with the CG group. After modelling, the frequency of gastric electrical stimulation in MG and four different MA groups was decreased (*P* < 0.05) when compared with the CG group. Four different MA manipulations increased frequency of gastric electrical stimulation at different time points, and all are reflective of the recovery of frequency in gastric electrical stimulation. The effect from FTG/RTG/FLG on the regulation of frequency is consistent.

Moreover, it is illustrated that FTG/RTG/FLG immediately shows an increase in frequency and then kept the improvement for five minutes after MA manipulation, during which the RTG and FLG groups even showed excessive frequency performance. Overall, the frequency of gastric electrical stimulation reflects a self-regulating trend of the acupuncture-induced rise and fall of frequency. It was shown, however, that the RLG group had a frequency decrease from the other three groups five minutes after MA manipulation, but at 20 minutes after MA manipulation, the frequency gradually recovered to the normal range. Future studies may examine the reasoning for the difference of RLG manipulation effects compared to the other styles, and the possible effects of increasing observational time and measurements for the FTG/RTG/FLG groups for a similar experiment.

Although both FTG and FLG are classified as reinforcing style (Bu) according to the principle of acupuncture manipulation, they should induce a reinforcement effect through the treatment of different diseases in the clinic. However, the physiological and biochemical effects on the body might be different, especially in terms of one specific index, in this study, gastric frequency or amplitude. According to the results, all four acupuncture manipulations achieved a benign adjustment of the gastric electrical frequency; however, the onset time between different manipulations are variable. In general, from the result at the During MA and After MA 20 min, we can see the effect of groups of the twisting manipulations gradually increases with time, while the effect of groups of the lifting-thrusting manipulations decreasing along with the time passing by. It seems that manipulations with similar movements are shown to be linked relatively closer on acupuncture effect.

What is more, this might also because of the acupuncture has the dual-directional regulatory effect, causing the reduction effect in an excessive model and the reinforcement effect in a deficiency model. In this study, the bradygastria rabbit is more likely a deficiency model, so the general trend of all manipulations is to facilitate the recovery of the gastric frequency.

Last but not least, we consider that different mechanoreceptors were activated by different manipulations. They tend to further activate different pressure units of the interstitial myofascial tissue receptors. Research has indicated that different acupuncture manipulations can give rise to distinct neural electrical codes [27, 28]. Different manipulations themselves cause the excitement of receptors in the afferent nervous system. This then stimulates the signal-induced integration and alternation of the electrical signals in the central nervous system and projects to various structures in the hypothalamus, the midbrain, and the medulla, so that the body may produce a variety of responses in multiple organ systems [29, 30]. In this study, manipulations on ST36 might excite the somato-parasympathetic reflex, which then involves afferent somatic nerves that go to the brain, and eventually the vagus nerve, followed by the change EGG, and ultimate release of gastrin and stimulation of gastrointestinal protein.

Thus, future studies should focus on which receptors are activated by different acupuncture manipulations, respectively, and in what ways these receptors amplify signals to generate different neural electrical signals.

## 5. Conclusion

The results of this study suggest that all four different acupuncture manipulations have a recovery effect on the gastric electrical frequency of rabbits with bradygastria. Among them, FTG, RTG, and FLG manipulations at acupoint ST36 could lead to a regular and significant recovery trend through the whole process of the acupuncture intervention.

## Data Availability

The data used to support the findings of this study are available from the first author upon request.
